# Validation of Trøndelag Apnoea Score Proxy for Obstructive Sleep Apnoea in the General Population of Norway: The HUNT Study

**DOI:** 10.1155/2024/1242505

**Published:** 2024-06-06

**Authors:** James Filosa, Petter Moe Omland, Knut Hagen, Knut Langsrud, Morten Engstrøm, Trond Sand

**Affiliations:** ^1^ Department of Neuromedicine and Movement Science Faculty of Medicine and Health Sciences Norwegian University of Science and Technology, Trondheim, Norway; ^2^ Department of Neurology and Clinical Neurophysiology St. Olavs Hospital Trondheim University Hospital, Trondheim, Norway; ^3^ Department of Radiology and Nuclear Medicine St. Olavs Hospital Trondheim University Hospital, Trondheim, Norway; ^4^ Norwegian Headache Research Centre (NorHEAD), Trondheim, Norway; ^5^ Clinical Research Unit Central Norway St. Olavs Hospital Trondheim University Hospital, Trondheim, Norway; ^6^ St. Olavs Hospital Trondheim University Hospital Østmarka, Trondheim, Norway

## Abstract

The aim was to validate a new seven-item “TASC” (Trøndelag Apnoea Score) proxy for obstructive sleep apnoea (OSA) against polysomnography in the general population. Objectives included validation against different polysomnographic criteria, stratification by age and gender, and estimation of OSA prevalence. From the fourth wave of the Trøndelag Health Study (HUNT4), 1,201 participants were randomly invited to a substudy focusing on sleep and headaches, of whom 232 accepted and 84 (64% women, mean age 55.0 years, and standard deviation 11.5 years) underwent polysomnography. The TASC proxy sums seven binary items for snoring, observed breathing pauses, restricted daytime activities, hypertension, body mass index (≥30 kg/m^2^), age (≥50 years), and gender (male). A single night of ambulatory (home) polysomnography was analysed using both the recommended and optional hypopnoea criteria of the American Academy of Sleep Medicine (AASM). We found 65% sensitivity and 87% specificity (Cohen's *κ* = 0.53, 95% confidence interval 0.34−0.72) for TASC ≥ 3 against AHI ≥ 15 (recommended AASM criteria). Validity was similar against AHI ≥ 30 but lower against AHI ≥ 5 and against the optional AASM criteria. Sensitivity and overall validity were higher among men and those above 50 years of age. The prevalence of an apnoea-hypopnoea index (AHI) of at least 5, 15, or 30 using the recommended (and optional) AASM criteria was 73% (46%), 37% (18%), or 15% (5%). A seven-item TASC proxy for OSA showed good validity and may be useful in screening and epidemiological settings. Sensitivity, specificity, and validity vary considerably by cut-off, by polysomnographic scoring criteria, and by gender and age strata.

## 1. Introduction

Obstructive sleep apnoea (OSA) is linked to a reduced quality of life, with unrefreshing sleep, sleepiness, fatigue, and depressive mood as potential mediators [1, 2]. It is strongly associated with adverse health conditions including atrial fibrillation, heart failure, stroke and coronary heart disease [3], and motor vehicle accidents [4]. It is therefore important to develop and validate proxy diagnoses for OSA in epidemiological studies.

The third edition of the International Classification of Sleep Disorders (ICSD-3), by the American Academy of Sleep Medicine (AASM), defines OSA by the number of predominantly obstructive, respiratory events per hour (apnoea-hypopnoea index (AHI), preferably by polysomnography (PSG)), symptoms, and comorbidities [2]. The criteria are met by AHI ≥ 15 alone but also by AHI ≥ 5 plus at least one of the following: snoring, breathing pauses, daytime symptoms of poor sleep (sleepiness, nonrestorative sleep, fatigue, or insomnia symptoms), or a diagnosis of a listed comorbidity (including hypertension). However, researchers typically operationalise the lone AHI cut-offs of 5, 15, and 30, referred to as “mild,” “moderate,” and “severe” OSA.

The prevalence of mild and moderate-to-severe OSA in the general population varies between 9% to 38% and 6% to 17%, respectively [5]. Prevalence is known to increase with body mass index (BMI), age, and male gender [2, 6], but it also doubles with the use of the recommended 2012 AASM criteria compared with 2007 AASM criteria [5, 7, 8]. Accordingly, there are concerns that both the symptoms and comorbidities accepted by the ICSD-3 and the latest AASM criteria for OSA are too inclusive. Two population-based studies among men above 40 years of age estimated the prevalence of ICSD-3 OSA at 52.2% (AHI ≥ 10, 2007 AASM criteria) and 74.4% (AHI ≥ 5, 2012 AASM criteria) [9, 10]. It is therefore of interest to estimate OSA prevalence by different AHI cut-offs and old versus new AASM criteria for hypopnoea. Also, OSA proxies have not previously been comparatively validated against the old and new AASM criteria, to our knowledge.

Although useful in epidemiological and some clinical settings, OSA proxies may never completely replace objective sleep testing [11]. The popular, eight-item STOP-Bang questionnaire was developed for surgical populations to rapidly evaluate the risk of OSA, being associated with perioperative complications [12]. It appeared superior to pre-existing questionnaires [13, 14] and has since been validated in numerous populations [15]. Since neck circumference measurements are rarely available in large epidemiological studies relying on questionnaires, including the fourth wave of the Trøndelag Health Study (HUNT4), it is of great interest to develop a STOP-Bang-inspired proxy without neck circumference.

While the STOP-Bang accepts any of tiredness, fatigue, or sleepiness as daytime symptoms of OSA, it is of interest of simplicity to settle on one daytime symptom of OSA. A recent large cohort study found only a weak association between the Epworth Sleepiness Scale (≥11) and the AHI [16], and excessive daytime sleepiness is less typical among women [17]. Meanwhile, elderly cases may have fewer symptoms altogether [18]. These differences may impact validity and necessitate gender- and age-specific proxy cut-offs [19]. Finally, the ICSD-3 states no minimum frequency of daytime symptoms. It is therefore of interest to validate a new proxy incorporating different daytime symptoms, of different frequencies, specified in the fifth edition of the Diagnostic and Statistical Manual of Mental Disorders (DSM-5) and the ICSD-3 (e.g., insomnia), in age and gender strata.

The general aim of this population-based study was to validate a new seven-item proxy for OSA, named the Trøndelag Apnoea Score (TASC), using items for snoring, breathing pauses, daytime symptoms, hypertension, BMI, age, and gender, against a PSG-based diagnosis, in an adult general population subsample from HUNT4 in Norway. The main objective was to validate several cut-offs of the TASC against AHI ≥ 5, AHI ≥ 15, and AHI ≥ 30, using the recommended AASM criteria. Secondary objectives were to study validity by the choice of daytime symptom (restricted daytime activities, sleepiness, or tiredness), study validity by the choice of minimum frequency of symptoms, stratify validity by age and gender strata, validate the TASC against the optional AASM criteria, and estimate prevalence of PSG- and proxy-based prevalence of OSA.

## 2. Methods

### 2.1. Participants

HUNT4 took place between August 2017 and February 2019. All residents above 20 years of age (meeting the World Health Organization criteria of adulthood) of the defunct Nord-Trøndelag county were invited to two questionnaires, a structured interview and a clinical examination [20], which 56,078 out of 96,469 residents (58%) underwent. Next, HUNT4 participants from Stjørdal municipality were invited by postal mail to an approved HUNT4 substudy named Sleep and Pain. Stjørdal municipality is a 938 km^2^ agricultural area with a small town centre and 23,165 inhabitants sufficiently representative of Trøndelag county. Out of 1,201 randomly selected HUNT4 participants, 232 (19%) agreed by telephone and were scheduled appointments at the interview site in Stjørdal in November 2017. They completed a waiting room questionnaire about sleep and health pending a face-to-face interview about sleep, health, headache, and pain. Finally, the participants of Sleep and Pain study were invited to a nerve conduction study (included for differential diagnostics of restless leg syndrome) and a single-night ambulatory PSG, which was initially accepted by 87 participants. However, two participants withdrew after the nerve conduction study, and the polysomnogram of another was omitted because of technical issues, resulting in 84 participants for the current PSG study.

The HUNT4 organisation performed the randomisation, and the regional ethics committee approved the invitation letter. The median time delay between questionnaire completion in the Sleep and Pain substudy and the PSG study was 11 months (range 3–22 months, 94% within 15 months). We documented clinical findings including advice for clinical follow-up in the electronic patient journal, sending copies to the participant and their general practitioner. Participants previously with diagnosed OSA (10 out of 232: 4%; 4 out of 84 with PSG: 5%) or any other disease were included. The validity of questionnaire-based diagnoses for insomnia, primary headaches, and restless leg syndrome has been published for this sample [21–23].

### 2.2. Questionnaires and Other Health-Related Data

In the Sleep and Pain substudy, participants completed the Karolinska Sleep Questionnaire (KSQ) [24] which includes items for snoring, apnoeas, and presumed daytime symptoms of sleep disturbances (Supplementary Table 1). Other questionnaires included the Epworth Sleepiness Scale [25], Insomnia Severity Index (ISI) [26], and the Hospital Anxiety and Depression Scale (HADS) [27]. Participants listed their health conditions, which were supplemented by their medication list. On the day of the ambulatory PSG, the height and weight of participants were measured using a wall-mounted height measuring tape and a bathroom-type body weight scale (SECA®, Hamburg, Germany), respectively.

### 2.3. The TASC Proxy

Inspired by the STOP-Bang [12], the TASC proxy dichotomises and sums seven OSA-relevant items. From the KSQ, loud and embarrassing snoring (according to others), breathing pauses during the night (according to others), and restricted daytime activities (spare time, school, or job) were each scored if reported “mostly/at least three times a week” (Supplementary Table 1). The remaining four items were hypertension (by questionnaire or list of medications), BMI above 30 kg/m^2^, age above 50 years, and male gender.

The main TASC proxy used restricted daytime activities as its daytime symptom of OSA because of its inclusion in the main HUNT4 questionnaire and its close resemblance to the DSM-5 insomnia diagnosis (criterion B). However, we also incorporated bothersome daytime sleepiness and bothersome daytime tiredness/fatigue into alternative proxies: “TASC-sleepy” and “TASC-tired.” Finally, a more liberal “TASC-monthly” allowed the three KSQ items to be reported “sometimes/at least once a month.”

### 2.4. PSG: Setup and OSA Diagnoses

The PSG equipment for the single night of unattended, ambulatory PSG was mounted at St. Olavs Hospital, Trondheim University Hospital, at 12:00 the preceding day. Participants were instructed to avoid alcohol, hypnotic drugs, and napping after dinner (unless done routinely), to go to sleep between 22:00 and 00:00 under undisturbed conditions (as normal), and to document the lights off and lights on time plus any awakenings (e.g., visits to the toilet). The equipment was dismantled at 08:00 the following morning.

The PSG was recorded using SOMNOscreen plus PSG equipment (SOMNOmedics GmbH®, Randersacker, Germany). Six electroencephalography (EEG) electrodes were placed according to the International 10-20 system: F3, F4, C3, C4, O1, and O2. Two electrooculographic electrodes were placed: 1 cm laterally and 2 cm above the right eye cantus and 1 cm laterally and 2 cm below the left eye cantus. Mastoid M1 and M2 were reference electrodes for electrooculographic and the contralateral EEG electrodes. Surface electromyography was registered from the submental and bilateral anterior tibial muscles. Nasal flow and naso-oral thermistor were affixed above the upper lip. Thoracic and abdominal piezoelectric respiratory effort belts were applied. Pulse oximetry was recorded from the index finger. The PSG was analysed using DOMINO® (version 3.0.2, SOMNOmedics).

As per the latest wording of the AASM manual (February 2023) [28], we used both the recommended (1a) and the optional (1b) hypopnoea criteria to calculate the AHI. The recommended hypopnoea criteria score a hypopnoea if there is an airflow signal drop of ≥30% for ≥10 seconds, associated with either an EEG arousal or a 3% oxygen desaturation. The more conservative, optional hypopnoea criteria instead require the hypopnoea to be associated with a 4% oxygen desaturation, disregarding any arousal. Our senior sleep expert calculated the AHI manually for each polysomnogram, first using the optional criteria and then (several months later) using the recommended criteria on raw PSG data, without any previously scored markers but not formally blinded to the initial scoring. For validation purposes, we used AHI cut-offs 5, 15, and 30. For prevalence estimation, we additionally assigned the ICSD-3 diagnosis to participants with AHI ≥ 5 plus at least one of the following: AHI ≥ 15, a complaint of snoring, breathing pauses, a daytime symptom (restricted activities, sleepiness, or tiredness; Table 1) or any insomnia symptom (difficulty falling asleep, falling back asleep, or waking up too early), at least three times a week, or any self-reported comorbidity listed by the ICSD-3 [2].

### 2.5. Statistics

Sensitivity, specificity, predictive values, and Cohen's kappa (*κ*) statistic [29] were calculated from two-by-two cross-tabulations of proxy cut-offs versus AHI cut-offs (recommended AASM criteria). The TASC cut-offs ≥2, ≥3, and ≥4 were explored in the main analysis, the results of which guided further analyses. Cohen's *κ* was interpreted as poor (*κ* ≤ 0.20), acceptable (0.20 ≤ *κ* ≤ 0.40), good (0.40 ≤ *κ* ≤ 0.60), very good (0.60 ≤ *κ* ≤ 0.80), or excellent (*κ* ≥ 0.80) overall validity [30]. Validity was stratified by age (below or above 50 years) and gender (women, men) and additionally calculated for the optional AASM criteria. Ninety-five percent confidence intervals (95% CI) for sensitivity, specificity, predictive values, and prevalence were calculated using the exact Clopper-Pearson method for binomial proportions [31]. The 95% CI for Cohen's *κ* used the asymptotic standard error generated by IBM SPSS®. We also produced some main receiver operating characteristic (ROC) curves. We used Microsoft Office Excel 2016 and IBM SPSS® version 28 to analyse the data.

Out of the 84 participants with complete PSG analyses, response rates for the three KSQ items regarding daytime symptoms were 100%. However, 10 participants (12%) failed to answer the item regarding breathing pauses, of whom four (5%) also failed to answer the item about loud and embarrassing snoring. For the primary analysis, the response option “never” was imputed for these 14 blank responses as to include all 84 participants. In a supplementary sensitivity analysis, we treated the blank responses to these questions as missing observations, initially leaving only 74 participants with determined proxy scores. For many of the proxy cut-offs however, these 10 participants could be classified as either definite positives or definite negatives owing to the score from the remaining five TASC items. Hence, the final sample size in the supplementary analysis varied between 78 and 82.

## 3. Results

### 3.1. Population Characteristics

Out of a total of 84 participants with complete PSG analyses, 55 (65%) were women and 60 (71%) were above 50 years of age (Table 1). The mean age of the total sample was 55.0 years, and the mean BMI was 27.2. Twenty-four percent of participants had a BMI above 30, 21% had hypertension, 21% was medicated for other cardiovascular diseases or risk factors, and 32% had DSM-5 insomnia by a diagnostic interview. Other comorbidities were less frequent (e.g., 5% diabetes, 2% asthma, 1% cancer history, and 8% polyneuropathy). The mean AHI of the total sample was 14.9 and 7.9 using the recommended and optional AASM criteria, respectively, being higher among men and participants above 50 years of age (Table 1).

The proportion of participants reporting KSQ items mostly/at least three times a week was 25% for loud and embarrassing snoring, 11% for breathing pauses, 11% for restricted daytime activities, 20% for daytime sleepiness, and 25% for daytime tiredness. Men reported more snoring and breathing pauses (Table 1). Three participants were omitted due to incomplete PSG analyses: two women and one man, all above 50 years of age. Their TASC scores were 1, 1, and 4, respectively. A further 10 participants had blank responses to questions concerning snoring or breathing pauses, of whom 10 had AHI ≥ 5, six had AHI ≥ 15, and one had AHI ≥ 30 (recommended AASM criteria).

### 3.2. Validity of the TASC Proxy (Recommended AASM Criteria)

**Figure 1:**
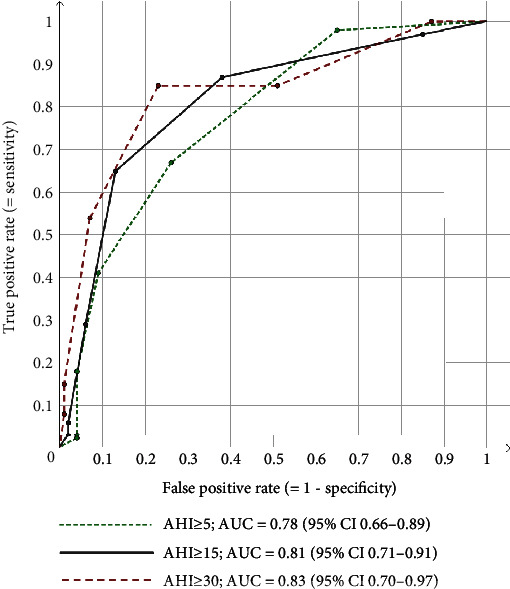
ROC curves of the TASC proxy against AHI categories (recommended AASM criteria). AHI: apnoea-hypopnoea index; AASM: American Academy of Sleep Medicine; ROC: receiver operating characteristic; AUC: area under the curve; CI: confidence interval; TASC (Trøndelag Apnoea Score): a seven-item OSA proxy with one potential point for each item: loud and embarrassing snoring (according to others) “mostly/at least three times a week,” breathing pauses during the night (according to others) “mostly/at least three times a week,” restricted daytime activities (spare time, school, or job) “mostly/at least three times a week,” hypertension, BMI ≥ 30, age ≥ 50 years, and male gender.

The optimal TASC cut-off was ≥2 against AHI ≥ 5, ≥3 against AHI ≥ 15, and ≥4 against AHI ≥ 30 (Table 2). However, TASC ≥ 2 showed only acceptable validity against AHI ≥ 5 (sensitivity = 67%, specificity = 74%, Cohen's *κ* = 0.35) while TASC ≥ 3 showed good validity against AHI ≥ 15 (sensitivity = 65%, specificity = 87%, Cohen's *κ* = 0.53), as did TASC ≥ 4 against AHI ≥ 30 (sensitivity = 54%, specificity = 93%, Cohen's *κ* = 0.48). Higher proxy cut-offs favoured specificity whereas higher AHI cut-offs favoured sensitivity. See Tables 2, 3, and 4 for predictive values. See Figure 1 for the associated ROC curves.

### 3.3. Validity by Daytime Symptom and Symptom Frequency (Recommended AASM Criteria)

**Figure 2:**
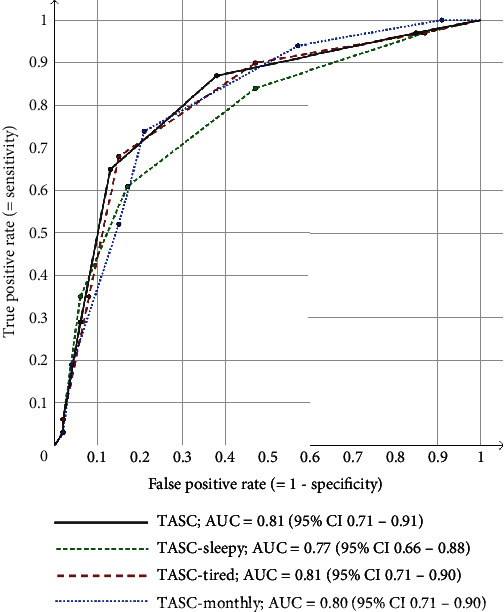
ROC curves of the TASC proxy, by daytime symptom and symptom frequency, against AHI ≥ 15 (recommended AASM criteria). AHI: apnoea-hypopnoea index; AASM: American Academy of Sleep Medicine; ROC: receiver operating characteristic; AUC: area under the curve; CI: confidence interval; TASC (Trøndelag Apnoea Score): a seven-item OSA proxy with one potential point for each item: loud and embarrassing snoring (according to others) “mostly/at least three times a week,” breathing pauses during the night (according to others) “mostly/at least three times a week,” restricted daytime activities (spare time, school, or job) “mostly/at least three times a week,” hypertension, BMI ≥ 30, age ≥ 50 years, and male gender; TASC-sleepy: requiring bothersome daytime sleepiness (as opposed to restricted daytime activities); TASC-tired: requiring bothersome daytime sleepiness (as opposed to restricted daytime activities); TASC-monthly: requiring the KSQ items “sometimes/at least once a month” (as opposed to “mostly/at least three times a week”).

The major difference between the alternative proxies was the higher sensitivity, but lower specificity, of the more liberal TASC-monthly (Table 3). TASC-sleepy and TASC-tired were only slightly more sensitive and less specific, compared to TASC. Still, all four proxies showed acceptable validity using proxy cut‐off ≥ 2 against AHI ≥ 5 (Cohen's *κ* 0.30−0.35), good validity using proxy cut‐off ≥ 3 against AHI ≥ 15 (Cohen's *κ* 0.45−0.53), and good validity using proxy cut‐off ≥ 4 against AHI ≥ 30 (Cohen's *κ* 0.43−0.51). See Figure 2 for the associated ROC curves.

### 3.4. Gender- and Age-Stratified Validity (Recommended AASM Criteria)

Against AHI ≥ 15, the optimal TASC cut-off was ≥2 among those below 50 years of age and women and ≥3 among those above 50 years of age and men (Table 4). Using these stratum-specific proxy cut-offs, TASC was slightly more sensitive for those above, versus below 50 years of age (69% vs. 60%), while similarly specific (82% vs. 84%), yielding slightly higher validity in the older age group (Cohen's *κ* = 0.52 vs. 0.41). TASC was also more sensitive among men, compared with women (88% vs. 73%), while similarly specific (69% vs. 75%), resulting in higher validity among men (Cohen's *κ* = 0.58 vs. 0.43).

### 3.5. Prevalence of OSA: ICSD-3, AHI Categories, and the TASC Proxy

Using the recommended AASM criteria, the prevalence of ICSD-3 OSA was 61% for the total sample, 53% among women, 76% among men, 42% among those below 50 years, and 68% among those above 50 years of age (Table 5). Using the recommended AASM criteria in the total sample, the prevalence of AHI ≥ 5, AHI ≥ 15, and AHI ≥ 30 was 73%, 37%, and 15%, respectively. The corresponding estimates using the optional AASM criteria were 46%, 18%, and 5%.

The prevalence of TASC ≥ 2, ≥3, and ≥4 was 56%, 32%, and 14%, respectively, in the total sample. Although TASC ≥ 2 produced the closest estimate to the ICSD-3 in the total sample, it overestimated prevalence among men (90% vs. 76%) and underestimated prevalence among women (38% vs. 53%) and those below 50 years of age (25% vs. 42%).

### 3.6. Validity against Optional AASM Criteria

Using the optional AASM criteria instead, the optimal cut-off for TASC was 3 against AHI ≥ 5 and AHI ≥ 15 and 4 against AHI ≥ 30 (Supplementary Table 2). Using TASC ≥ 3 against AHI ≥ 15, sensitivity was higher (73% vs. 65%), but specificity was lower (77% vs. 87%), than with the use of recommended AASM criteria, and validity was only acceptable (Cohen's *κ* 0.38 vs. 0.53).

### 3.7. Validity of the TASC Proxy, excluding Blank Respondents to Snoring and Breathing Pauses (Recommended AASM Criteria)

Excluding blank respondents to snoring and breathing pauses (according to others), instead of imputing missing responses, yielded slightly higher sensitivity and validity but similar validity overall (Cohen's *κ* 0.33−0.58, Supplementary Table 3).

## 4. Discussion

In this population-based sample, we found good validity (65% sensitivity, 87% specificity, Cohen's *κ* = 0.53) of a seven-item STOP-Bang-inspired proxy for OSA (TASC), using the cut‐off ≥ 3, against PSG-based AHI ≥ 15 (recommended AASM scoring criteria). Validity was similar against AHI ≥ 30, but mostly acceptable against AHI ≥ 5. There were minimal differences when incorporating different, alternative daytime symptoms into the TASC proxy. Sensitivity and overall validity were higher among men compared with women and in those above versus below 50 years of age. Validity was only acceptable using the conservative optional AASM criteria. Using the recommended AASM criteria, the prevalence of AHI ≥ 5, AHI ≥ 15, and AHI ≥ 30 was 73%, 37%, and 15%, versus 46%, 18%, and 5%, using the more conservative optional criteria. The prevalence of ICSD-3 OSA was 61% with the recommended and 37% with the optional AASM criteria. TASC ≥ 3 was reasonably prevalent in this sample, at 32% overall.

### 4.1. Comparisons with Other Validation Studies

In a recent systematic review and meta-analysis, Chen et al. [32] identified five validation studies of the STOP-Bang in the general population [14, 33–36]. Against AHI ≥ 5, AHI ≥ 15, and AHI ≥ 30, the authors estimated pooled sensitivity at 73%, 88%, and 92% and pooled specificity at 66%, 42%, and 38%, respectively. Given the pooled prevalence rates, this corresponds to Cohen's *κ* estimates of merely 0.39 against AHI ≥ 5, 0.17 against AHI ≥ 15, and 0.07 against AHI ≥ 30. While we found similar estimates of sensitivity and specificity, we found considerably higher validity overall (Cohen's *κ* = 0.41−0.53). However, one should note that the STOP-Bang cut-off was fixed at ≥3 while we let TASC cut-off varies between ≥2 and ≥4. Considering that three out of the five identified studies [14, 33, 35] used a 4% desaturation threshold only to score hypopnoea, the pooled results should perhaps be compared to our results against the optional AASM criteria instead, which indicated lower validity (Cohen's *κ* = 0.33−0.38).

Beyond the proxy cut-off, comparisons with the pooled results are hindered by the use of type 3 devices (without sleep and EEG arousal scoring) in two studies [33, 36], which have roughly 90% sensitivity and specificity against the gold-standard PSG [37]. Given an underlying relationship between questionnaire scores and the PSG, the use of type 3 devices introduces nondifferential misclassification of cases and noncases which will weaken the observed STOP-Bang validity. In all, our present TASC scores appear more valid than the STOP-Bang although there are few population-based studies using the gold-standard PSG [14, 34].

Whereas the STOP-Bang only requires the symptom frequency “often” for daytime tiredness, fatigue, or sleepiness (T) [12], the current KSQ-based TASC proxy specifies a symptom frequency of “mostly/at least three times a week” for snoring, breathing pauses, and daytime symptoms. We found no advantage of the more relaxed frequency criteria “sometimes/at least once a month” on overall validity, against any AHI cut-off, using any set of AASM criteria. Considering the focus on frequency criteria, the current proxies may also be likened to the Berlin Questionnaire [38]. It focuses on the “STOP” items (particularly snoring and tiredness) and requires a frequency of “nearly every day” or “3−4 times a week” for five out of 10 items, congruent with our main KSQ response option of “mostly/at least three times a week.” In a systematic review, Senaratna et al. [39] identified two validation studies of the Berlin Questionnaire in the general population. Hrubos-Strøm et al. [40] found only 37% sensitivity and 84% specificity (Cohen's *κ* = 0.20) against AHI ≥ 5 and 43% sensitivity and 80% specificity (Cohen's *κ* = 0.13) against AHI ≥ 15, using a 4% desaturation threshold for hypopnoea scoring. Meanwhile, Kang et al. [41] found 69% sensitivity and 83% specificity (Cohen's *κ* = 0.48) against AHI ≥ 5 and 89% sensitivity and 63% specificity (Cohen's *κ* = 0.40) against AHI ≥ 15, using a 3% threshold to score hypopnoeas. Hence, from the few available studies in the general population, the explicit use of a minimum symptom frequency (Berlin Questionnaire and the current TASC) may be an improvement on the vaguer wording of the STOP-Bang.

Marti-Soler et al. [34] derived and optimised a five-item score called NoSAS to a large population-based sample. Against AHI ≥ 20, the NoSAS outperformed both the STOP-Bang and Berlin Questionnaire in two separate cohorts (Cohen's *κ* = 0.37−0.39 vs. 0.15−0.22). This difference may not be completely attributed to the use of predefined cut-offs for the STOP-Bang and Berlin Questionnaire, as the NoSAS also had a greater area (0.74−0.81 vs. 0.63−0.68) under the ROC curve.

The validity of the current TASC ≥ 3 may also be compared with that of proxies for interview-verified headache and sleep disorder diagnoses in the same sample [21–23]. As judged by Cohen's *κ*, TASC ≥ 3 performed similarly to proxies for headache suffering, migraine, insomnia, and unspecified restless leg syndrome (Cohen's *κ* = 0.45−0.57) and better than the proxy for tension-type headache (Cohen's *κ* = 0.33).

### 4.2. Items and Strata

There were minimal differences in validity between the different daytime symptoms. The agreement proportion between any two TASC proxies (≥3) with different daytime symptoms (restricted activities, sleepiness, or tiredness) was at least 96% (Cohen's *κ* ≥ 0.92, not tabulated), partially because 72% to 89% of participants with TASC ≥ 3 did not report the targeted symptom at least three times a week. The choice of daytime symptom may then seem insignificant, but the agreement proportion of the three daytime symptoms was comparatively low, at 86% (Cohen's *κ* 0.46−0.59). Hence, the choice of daytime symptom may have a larger effect on simpler proxies that are less reliant on other items and on proxies that require a lower minimum frequency. We advocate restricted daytime activities as the most clinically relevant daytime symptom of OSA, given both its concordance with the DSM-5 diagnosis of insomnia and recent studies reporting weak associations between OSA and daytime sleepiness [16, 42]. Restricted daytime activities may also be partially viewed as the result of daytime tiredness, sleepiness, or fatigue.

Altogether, 63% to 72% of participants with OSA (depending on the proxy) reported at least one symptom (tiredness, snoring, or breathing pauses) at least three times a week. This contrasts with another population-based study, in the middle age, in which only a minority of participants with moderate-to-severe OSA reported symptoms [42]. The discrepancy may be due to the authors' use of the Epworth Sleepiness Scale, known to correlate poorly with the AHI [16], and highlights the need to standardise symptom evaluation by OSA proxies. Note that proxy-positive participants include asymptomatic cases, as a TASC score of four can be obtained from hypertension, BMI, age, and gender. The fact that asymptomatic cases do not necessarily benefit from treatment [43] deems such proxy scores more suitable to epidemiological studies than to clinical decision-making.

While the STOP-Bang uses BMI ≥ 35, we chose BMI ≥ 30 for better suitability to our population-based sample with a mean BMI of 27.2. Similarly, previous studies have found the optimal BMI cut-off to depend on ethnicity and gender, down to 30 for women [19] and as low as 27.5 in certain populations [44]. However, there is a growing concern that BMI fails to capture adiposity in all demographics. Some studies have used the waist-to-hip ratio as an alternative among women [45]. Perhaps the limitations of the BMI partially explain why our TASC proxy was more valid among men than women. Using health care use as a clinical endpoint, a large longitudinal study among persons aged between 45 and 85 years (age range of 85% of our sample) found similar risks whether obesity was defined by BMI, waist circumference, waist-hip-ratio, or body fat percentage [46]. The correlation between obesity and health care use was however weaker in the higher age categories [46].

Snoring and breathing pauses according to a bed partner may need special consideration since many participants lack a bed partner. As the 10 blank respondents had a higher AHI overall, we found slightly higher validity when not imputing these responses to “never.” Perhaps the lack of a bed partner should be viewed as a marker of poorer health, including greater risk of OSA. In a supplementary analysis (not tabulated), we explored six-item proxies by successively removing one of the 7 TASC items. Notably, the removal of breathing pauses slightly increased the estimate of validity (Cohen's *κ* = 0.55), while the removal of snoring or BMI slightly decreased the estimate of validity (Cohen's *κ* 0.48 and 0.45, respectively).

We found higher validity among those above (vs. below) 50 years of age. By comparison, we previously found lower validity of questionnaire-based diagnoses for insomnia and restless leg syndrome among elderly participants in the same population, proposing age-dependent decreases in reading comprehension or increases in competing causes of symptoms as potential mechanisms [21, 22]. Both these factors may have been weakened by the current inclusion of nonquestionnaire items and by the lower age cut-off in the current study (≥50 vs. ≥65 years). We also found the TASC to be more sensitive and more valid overall, among men compared with women, similarly to Bauters et al. [33]. The positive relation between validity and both age and male gender is evident from the stratified summary of the PSG (Table 1), in which older participants and men (in particular) show more obstructive sleep than their counterparts.

### 4.3. The Choice of Proxy Cut-Off

While we let Cohen's *κ* compare the overall validity of different proxy cut-offs, the intended application must also be taken into account. TASC ≥ 3 (sensitivity = 65%, specificity = 87%, Cohen's *κ* = 0.53) may be optimal for correlation studies wherein specificity is key, as to not dilute identified cases with false positives. Conversely, TASC ≥ 2 (sensitivity = 87%, specificity = 62%, Cohen's *κ* = 0.45) may be more suitable in screening settings, although OSA proxies are deemed unfit to replace objective sleep testing in the clinical setting [11]. Regarding prevalence estimation, one may be tempted to choose the cut-off that produces the closest prevalence estimate to the gold-standard reference in the validation study. However, Diggle [47] has shown that the proxy prevalence (and its closeness to the gold-standard) depends on the interplay between sensitivity, specificity, and the gold-standard prevalence itself. Hence, the optimal proxy cut-off for prevalence estimation should be based on overall validity rather than on the closeness in prevalence between proxy and gold-standard in a given study. In our study, TASC ≥ 2 produced the closest prevalence estimate to the ICSD-3 diagnosis and AHI ≥ 5, while TASC ≥ 3 was the closest to AHI ≥ 15 (recommended AASM criteria).

### 4.4. The Choice of AHI Cut-Off

The prevalence of OSA varied greatly with the choice of AASM (or ICSD-3) criteria and AHI cut-off and between gender and age categories. The particularly high prevalence of AHI ≥ 5 (and ICSD-3 OSA), at 73% with recommended AASM criteria, raises questions about its clinical and epidemiological relevance, in this population at least. The issue partly remains for AHI ≥ 5 using the more conservative, optional AASM criteria (46% prevalence). We therefore suggest greater relevance of AHI ≥ 15 than AHI ≥ 5, using the latest recommended AASM criteria.

The choice of AHI cut-off also affected the balance between sensitivity and specificity of our OSA proxies (at a fixed proxy cut-off). Compared with AHI ≥ 15, the proxies were more specific (less sensitive) against AHI ≥ 5 and more sensitive (less specific) against AHI ≥ 30, a trend also seen in previous validation studies [32]. Although a formal mathematical proof of this relation is beyond the scope of this study, one should note that changes in the AHI cut-off and the proxy cut-off have opposite effects on the balance between sensitivity and specificity.

### 4.5. Strengths and Limitations

A major strength of this study is its population-based recruitment of 1,201 HUNT4 participants. However, the sequential recruitment of participants via those who underwent the interview [21–23], and the joint invitation to the PSG study and a nerve conduction study, may have lowered the participation rate to 7% out of the initial 1,201 HUNT4 participants. By comparison, we achieved an 18% participation rate for ambulatory PSG alone in the HUNT3 PSG study [48]. Selection bias is most evident in the over-representation of women, elderly, and persons with insomnia (interview focus). On the other hand, an enrichment of subjects with sleep health issues ensured an adequate number of OSA cases from the 84 PSGs.

Regarding the PSG procedure itself, one major strength was the analysis using both the recommended and the optional AASM criteria. While there was a considerable delay between questionnaire completion and the PSG for many participants, sleep questionnaires like the Pittsburgh Sleep Quality Index seem to be reliable across several months [49], hypertension and untreated OSA can be considered stable traits (OSA prevalence increasing very slowly until age 65) [5], and BMI was calculated during the PSG setup. Although the AHI is known to between consecutive nights, suggesting repeated PSGs for a clinical diagnosis [50], estimated AHI night-to-night reliability is high in most studies [51]. Using a single night of PSG might be considered a weakness, but the so-called “first night effect” appears minimal for ambulatory PSG recordings [52, 53].

## 5. Conclusion

In this population-based sample, we found good validity of a new seven-item TASC proxy for OSA, against AHI ≥ 15 using a PSG-based gold-standard with the recommended AASM criteria. Validity was similar against AHI ≥ 30, but lower against AHI ≥ 5 and against the more conservative, optional, AASM criteria.

Sensitivity and overall validity were higher among men compared with women and in those above versus below 50 years of age. A seven-item TASC proxy for OSA should accordingly be useful in epidemiological studies. Researchers and clinicians should note how sensitivity, specificity, and validity vary by cut-off, polysomnographic criteria, and demographic strata.

## Figures and Tables

**Table 1:** Population characteristics.

	Total, *n* = 84	Age < 50 years, *n* = 24	Age ≥ 50 years, *n* = 60	Women, *n* = 55	Men, *n* = 29
Age, years: mean (SD) [range]	55.0 (11.5) [27–83]	41.3 (7.3)	60.5 (7.7)	54.2 (12.1)	56.7 (10.3)
BMI: mean (SD)	27.2 (4.3)	27.1 (4.9)	27.3 (4.1)	26.8 (4.8)	28.1 (3.2)
BMI ≥ 30: % (95% CI)	24 (15–34)	13 (3–32)	28 (17–41)	22 (12–35)	28 (13–47)
Daily/occasional smoker: % (95% CI)	38 (28–49)	46 (26–67)	35 (23–48)	44 (30–58)	28 (13–47)
Alcohol use >1 day per week: % (95% CI)	29 (19–40)	17 (5–37)	34 (22–47)	26 (15–40)	34 (18–54)
Married: % (95% CI)	54 (42–65)	33 (16–55)	62 (48–74)	50 (36–64)	61 (41–78)
Employed: % (95% CI)	65 (54–76)	83 (63–95)	58 (45–71)	64 (50–76)	69 (49–85)
Exercise at least twice a week: % (95% CI)	78 (68–87)	83 (63–95)	76 (63–86)	80 (66–89)	76 (56–90)
Education distribution (%): basic–high school–university	18–29–54	0–38–63	25–25–50	22–35–44	10–17–72
Hypertension: % (95% CI)	21 (13–32)	13 (3–32)	25 (15–38)	16 (8–29)	31 (15–51)
Other cardiovascular diseases (medicated): % (95% CI)	21 (13–32)	4 (0–21)	28 (17–41)	18 (9–31)	28 (13–47)
DSM-5 insomnia: % (95% CI)	32 (22–43)	25 (10–47)	35 (23–48)	38 (25–52)	21 (8–40)

*Summary of polysomnography: mean (SD)*					
AHI, recommended	14.9 (14.5)	10.4 (15.4)	16.7 (13.9)	11.7 (12.3)	20.9 (16.6)
AHI, optional	7.9 (10.2)	5.3 (9.7)	9.0 (10.2)	6.4 (8.5)	10.8 (12.3)
ODI, 3% threshold	14.4 (17.4)	14.5 (26.3)	14.4 (12.4)	11.9 (17.8)	19.2 (15.8)
ODI, 4% threshold	7.5 (10.6)	6.2 (11.9)	8.1 (10.0)	5.6 (8.3)	11.3 (13.3)
Sleep latency (minutes)	12 (18)	7 (6)	14 (21)	13 (18)	9 (18)
Total sleep time (hours)	6.4 (1.3)	6.9 (1.2)	6.2 (1.3)	6.4 (1.3)	6.4 (1.2)
Sleep efficiency: %	86 (15)	92 (8)	83 (16)	85 (17)	88 (11)

*Questionnaire scores: 25^th^–50^th^–75^th^ percentiles*					
Epworth Sleepiness Scale (max. 24)	3–5–8.75	3–5.5–10	3–5–8	3–5–9	4–6–8.5
Insomnia Severity Index (max. 28)	3.25–9–15	4–9–14.25	3–9.5–16	4–10–16	2–6–14.5
HADS-Anxiety (max. 21)	2–4–7	1.25–4.5–7.75	2–4–7	2–5–7	1–3–7
HADS-Depression (max. 21)	1–2–5	1–2.5–5.75	1–2–5	1–2–5	1–2–5

*KSQ items, distribution (%) of responses: never–rarely/a few times a year–sometimes/at least once a month–mostly/at least three times a week*					
Loud and embarrassing snoring (according to others)^∗^	25–27–23–25	29–21–25–25	23–30–22–25	33–29–15–24	10–24–38–28
Breathing pauses during the night (according to others)^∗^	81–7–1–11	83–8–0–8	80–7–2–12	87–5–2–5	69–10–0–21
Sleep difficulties restrict my daytime activities (spare time, school, or job)^∗^	40–25–24–11	25–33–33–8	47–22–20–12	35–25–25–15	52–24–21–3
Bothersome sleepiness during the day	14–35–31–20	4–29–46–21	18–37–25–20	16–27–31–25	10–48–31–10
Bothersome tiredness during the day	6–29–40–25	0–29–46–25	8–28–38–25	4–25–38–33	10–34–45–10

SD: standard deviation; BMI: body mass index; DSM-5: Diagnostic and Statistical Manual of Mental Disorders, Fifth Edition; AHI: apnoea-hypopnoea index; recommended/optional: using the recommended/optional scoring criteria from the American Academy of Sleep Medicine (see text); ODI: oxygen desaturation index; HADS: Hospital Anxiety and Depression Scale; KSQ: Karolinska Sleep Questionnaire. ^∗^KSQ items included in the main TASC (Trøndelag Apnoea Score) proxy (see Methods).

**Table 2:** Validity of the TASC proxy (recommended AASM criteria).

	Sensitivity: % (95% CI)	Specificity: % (95% CI)	Cohen's *κ* (95% CI)	Positive predictive value: % (95% CI)	Negative predictive value: % (95% CI)
*AHI* ≥ 5*(73% prevalence)*					
TASC ≥ 2	67 (54–79)	74 (52–90)	0.35 (0.15–0.54)	87 (74–95)	46 (29–63)
TASC ≥ 3	41 (29–54)	91 (72–99)	0.22 (0.09–0.36)	93 (76–99)	37 (24–51)
TASC ≥ 4	18 (9–30)	96 (78–100)	0.08 (0.00–0.17)	92 (62–100)	31 (20–43)

*AHI* ≥ 15*(37% prevalence)*					
TASC ≥ 2	87 (70–96)	62 (48–75)	0.45 (0.27–0.62)	57 (42–72)	89 (75–97)
TASC ≥ 3	65 (45–81)	87 (75–95)	0.53 (0.34–0.72)	74 (54–89)	81 (68–90)
TASC ≥ 4	29 (14–48)	94 (84–99)	0.27 (0.08–0.46)	75 (43–95)	69 (57–80)

*AHI* ≥ 30*(15% prevalence)*					
TASC ≥ 2	85 (55–98)	49 (37–61)	0.16 (0.03–0.30)	23 (12–38)	95 (82–99)
TASC ≥ 3	85 (55–98)	77 (66–87)	0.43 (0.23–0.63)	41 (22–61)	96 (88–100)
TASC ≥ 4	54 (25–81)	93 (84–98)	0.48 (0.22–0.75)	58 (28–85)	92 (83–97)

OSA: obstructive sleep apnoea; AHI: apnoea-hypopnoea index; AASM: American Academy of Sleep Medicine; TASC (Trøndelag Apnoea Score): a seven-item OSA proxy with one potential point for each item: loud and embarrassing snoring (according to others) “mostly/at least three times a week,” breathing pauses during the night (according to others) “mostly/at least three times a week,” restricted daytime activities (spare time, school, or job) “mostly/at least three times a week,” hypertension, BMI ≥ 30, age ≥ 50 years, and male gender. See Figure 1 for receiver operating characteristic curves.

**Table 3:** Validity of the TASC proxy by daytime symptom and symptom frequency (recommended AASM criteria).

	Sensitivity: % (95% CI)	Specificity: % (95% CI)	Cohen's *κ* (95% CI)	Positive predictive value: % (95% CI)	Negative predictive value: % (95% CI)
*AHI* ≥ 5*(73% prevalence)*					
TASC ≥ 2	67 (54–79)	74 (52–90)	0.35 (0.15–0.54)	87 (74–95)	46 (29–63)
TASC‐sleepy ≥ 2	70 (57–81)	65 (43–84)	0.31 (0.11–0.52)	84 (71–93)	45 (28–64)
TASC‐tired ≥ 2	74 (61–84)	65 (43–84)	0.35 (0.14–0.56)	85 (72–93)	48 (30–67)
TASC‐monthly ≥ 2	79 (66–88)	52 (31–73)	0.30 (0.08–0.52)	81 (69–90)	48 (28–69)

*AHI* ≥ 15*(37% prevalence)*					
TASC ≥ 3	65 (45–81)	87 (75–95)	0.53 (0.34–0.72)	74 (54–89)	81 (68–90)
TASC‐sleepy ≥ 3	61 (42–78)	83 (70–92)	0.45 (0.25–0.65)	68 (48–84)	79 (66–88)
TASC‐tired ≥ 3	68 (49–83)	85 (72–93)	0.53 (0.35–0.72)	72 (53–87)	82 (69–91)
TASC‐monthly ≥ 3	74 (55–88)	79 (66–89)	0.52 (0.34–0.71)	68 (49–83)	84 (71–93)

*AHI* ≥ 30*(15% prevalence)*					
TASC ≥ 4	54 (25–81)	93 (84–98)	0.48 (0.22–0.75)	58 (28–85)	92 (83–97)
TASC‐sleepy ≥ 4	62 (32–86)	92 (83–97)	0.51 (0.26–0.76)	57 (29–82)	93 (84–98)
TASC‐tired ≥ 4	62 (32–86)	90 (81–96)	0.49 (0.24–0.74)	53 (27–79)	93 (84–98)
TASC‐monthly ≥ 4	77 (46–95)	80 (69–89)	0.43 (0.21–0.64)	42 (22–63)	95 (86–99)

OSA: obstructive sleep apnoea; AHI: apnoea-hypopnoea index; AASM: American Academy of Sleep Medicine; TASC (Trøndelag Apnoea Score): a seven-item OSA proxy with one potential point for each item: loud and embarrassing snoring (according to others) “mostly/at least three times a week,” breathing pauses during the night (according to others) “mostly/at least three times a week,” restricted daytime activities (spare time, school, or job) “mostly/at least three times a week,” hypertension, BMI ≥ 30, age ≥ 50 years, and male gender; TASC-sleepy: requiring bothersome daytime sleepiness (as opposed to restricted daytime activities); TASC-tired: requiring bothersome daytime sleepiness (as opposed to restricted daytime activities); TASC-monthly: requiring the KSQ items “sometimes/at least once a month” (as opposed to “mostly/at least three times a week”). See Figure 2 for receiver operating characteristic curves for AHI ≥ 15.

**Table 4:** Gender- and age-stratified validity of the TASC proxy (recommended AASM criteria).

	Proxy	Sensitivity: % (95% CI)	Specificity: % (95% CI)	Cohen's *κ* (95% CI)	Positive predictive value: % (95% CI)	Negative predictive value: % (95% CI)
*AHI* ≥ 15*(37% prevalence)*						
Age < 50 years, *n* = 24	TASC ≥ 2	60 (15–95)	84 (60–97)	0.41 (-0.01–0.84)	50 (12–88)	89 (65–99)
	TASC ≥ 3	40 (5–85)	95 (74–100)	0.41 (-0.06–0.87)	67 (9–99)	86 (64–97)
Age ≥ 50 years, *n* = 60	TASC ≥ 2	92 (75–99)	50 (32–68)	0.40 (0.20–0.59)	59 (42–74)	89 (67–99)
	TASC ≥ 3	69 (48–86)	82 (65–93)	0.52 (0.30–0.74)	75 (53–90)	78 (61–90)
Women, *n* = 55	TASC ≥ 2	73 (45–92)	75 (59–87)	0.43 (0.18–0.67)	52 (30–74)	88 (73–97)
	TASC ≥ 3	40 (16–68)	93 (80–98)	0.37 (0.09–0.65)	67 (30–93)	80 (66–91)
Men, *n* = 29	TASC ≥ 2	100 (79–100)	23 (5–54)	0.25 (0.00–0.46)	62 (41–80)	100 (29–100)
	TASC ≥ 3	88 (62–98)	69 (39–91)	0.58 (0.28–0.87)	78 (52–94)	82 (48–98)

OSA: obstructive sleep apnoea; AHI: apnoea-hypopnoea index; AASM: American Academy of Sleep Medicine; TASC (Trøndelag Apnoea Score): a seven-item OSA proxy with one potential point for each item: loud and embarrassing snoring (according to others) “mostly/at least three times a week,” breathing pauses during the night (according to others) “mostly/at least three times a week,” restricted daytime activities (spare time, school, or job) “mostly/at least three times a week,” hypertension, BMI ≥ 30, age ≥ 50 years, and male gender.

**Table 5:** Prevalence of OSA: ICSD-3, AHI categories, and the TASC proxy: % (95% CI).

	Total, *n* = 84	Age < 50 years, *n* = 24	Age ≥ 50 years, *n* = 60	Women, *n* = 55	Men, *n* = 29
*ICSD-3 OSA*					
Recommended AASM criteria	61 (49–71)	42 (22–63)	68 (55–80)	53 (39–66)	76 (56–90)
Optional AASM criteria	37 (27–48)	21 (7–42)	43 (31–57)	31 (19–45)	48 (29–67)

*AHI categories, recommended AASM criteria*					
AHI ≥ 5	73 (62–82)	46 (26–67)	83 (71–92)	65 (51–78)	86 (68–96)
AHI ≥ 15	37 (27–48)	21 (7–42)	43 (31–57)	27 (16–41)	55 (36–74)
AHI ≥ 30	15 (9–25)	13 (3–32)	17 (8–29)	11 (4–22)	24 (10–44)

*AHI categories, optional AASM criteria*					
AHI ≥ 5	46 (35–58)	25 (10–47)	55 (42–68)	42 (29–56)	55 (36–74)
AHI ≥ 15	18 (10–28)	13 (3–32)	20 (11–32)	11 (4–22)	31 (15–51)
AHI ≥ 30	5 (1–12)	4 (0–21)	5 (1–14)	4 (0–13)	7 (1–23)

*OSA proxies*					
TASC ≥ 2	56 (45–67)	25 (10–47)	68 (55–80)	38 (25–52)	90 (73–98)
TASC ≥ 3	32 (22–43)	13 (3–32)	40 (28–53)	16 (8–29)	62 (42–79)
TASC ≥ 4	14 (8–24)	4 (0–21)	18 (10–30)	9 (3–20)	24 (10–44)

OSA: obstructive sleep apnoea; ICSD-3: International Classification of Sleep Disorders, 3^rd^ Edition; AHI: apnoea-hypopnoea index; AASM: American Academy of Sleep Medicine; TASC (Trøndelag Apnoea Score): a seven-item OSA proxy with one potential point for each item: loud and embarrassing snoring (according to others) “mostly/at least three times a week,” breathing pauses during the night (according to others) “mostly/at least three times a week,” restricted daytime activities (spare time, school, or job) “mostly/at least three times a week,” hypertension, BMI ≥ 30, age ≥ 50 years, and male gender.

## Data Availability

Data is available on request from the authors.

## Supplementary Materials

Supplementary Materials Supplementary Table 1: Karolinska Sleep Questionnaire items used for the TASC proxy and three TASC proxy variants (“-sleepy,” “-tired,” and “-sometimes”). Supplementary Table 2: validity of the TASC proxy against optional AASM criteria. Supplementary Table 3: validity of the TASC proxy against recommended AASM criteria: excluding blank respondents to snoring and breathing pauses.
