# Supplementary material for: Validation of Trøndelag Apnoea Score Proxy for Obstructive Sleep Apnoea in the General Population of Norway: The HUNT Study
Source: Sleep Disord. 2024 Jun 6;2024:1242505. doi: 10.1155/2024/1242505 (PMC11222008; doi:10.1155/2024/1242505)
Supplement: Supplementary Materials — Supplementary Table 1: Karolinska Sleep Questionnaire items used for the TASC proxy and three TASC proxy variants (“-sleepy,” “-tired,” and “-sometimes”). Supplementary Table 2: validity of the TASC proxy against optional AASM criteria. Supplementary Table 3: validity of the TASC proxy against recommended AASM criteria: excluding blank respondents to snoring and breathing pauses. [file 1242505.f1.docx]

# Supplementary Table 1: Karolinska Sleep Questionnaire items used for the TASC proxy and three TASC proxy variants (”-sleepy”, “-tired” and “-sometimes”)

| How OFTEN have you noticed the following during the last 3 months? (Put one cross for each question in the table) | | | | |
| --- | --- | --- | --- | --- |
|  | **never** | **rarely,**  a few times a year | **sometimes,**  at least once a month | **mostly,**  at least three times a week |
| Loud and embarrassing snoring (according to others) |  |  | TASC-sometimes | TASC  TASC-sleepy  TASC-tired |
| Breathing pauses during the night (according to others) |  |  | TASC-sometimes | TASC  TASC-sleepy  TASC-tired |
| Sleep difficulties restrict my daytime activities (spare time, school or job) |  |  | TASC-sometimes | TASC |
| Bothersome sleepiness during the day |  |  |  | TASC-sleepy |
| Bothersome tiredness during the day |  |  |  | TASC-tired |

TASC: Trøndelag Apnoea Score. Recall that all four variants of the TASC proxy also incorporate four non-questionnaire variables: hypertension, BMI≥30, age≥50 years, and male gender.

# Supplementary Table 2: Validity of the TASC proxy against optional AASM criteria

|  | **sensitivity:**  **% (95% CI)** | **specificity:**  **% (95% CI)** | **Cohen’s κ**  **(95% CI)** | **positive predictive value:**  **% (95% CI)** | **negative predictive value:**  **% (95% CI)** |
| --- | --- | --- | --- | --- | --- |
|  | **AHI≥5 (46% prevalence)** | | | | |
| TASC≥2 | 74 (58−87) | 60 (44−74) | 0.34 (0.14−0.54) | 62 (46−75) | 73 (56−86) |
| TASC≥3 | 51 (35−68) | 84 (71−94) | 0.36 (0.17−0.56) | 74 (54−89) | 67 (53−79) |
| TASC≥4 | 26 (13−42) | 96 (85−99) | 0.22 (0.06−0.38) | 83 (52−98) | 60 (47−71) |
|  | **AHI≥15 (18% prevalence)** | | | | |
| TASC≥2 | 87 (60−98) | 51 (38−63) | 0.20 (0.06−0.35) | 28 (16−43) | 95 (82−99) |
| TASC≥3 | 73 (45−92) | 77 (65−86) | 0.38 (0.17−0.59) | 41 (22−61) | 93 (83−98) |
| TASC≥4 | 33 (12−62) | 90 (80−96) | 0.25 (-0.01−0.51) | 42 (15−72) | 86 (76−93) |
|  | **AHI≥30 (5% prevalence)** | | | | |
| TASC≥2 | 100 (40−100) | 46 (35−58) | 0.08 (0.00−0.15) | 9 (2−20) | 100 (91−100) |
| TASC≥3 | 100 (40−100) | 71 (60−81) | 0.19 (0.03−0.36) | 15 (4−34) | 100 (94−100) |
| TASC≥4 | 75 (19−99) | 89 (80−95) | 0.33 (0.03−0.63) | 25 (5−57) | 99 (93−100) |

OSA: Obstructive Sleep Apnoea. AHI: Apnoea-Hypopnoea Index. AASM: American Academy of Sleep Medicine.

TASC (Trøndelag Apnoea Score): a seven-item OSA proxy with one potential point for each item:

loud and embarrassing snoring (according to others) “mostly/at least three times a week”; breathing pauses during the night (according to others) “mostly/at least three times a week”; restricted daytime activities (spare time, school or job) “mostly/at least three times a week”; hypertension; BMI≥30; age≥50 years; male gender.

# Supplementary Table 3: Validity of the TASC proxy, excluding blank respondents to snoring and breathing pauses (recommended AASM criteria)

|  | **sensitivity:**  **% (95% CI)** | **specificity:**  **% (95% CI)** | **Cohen’s κ**  **(95% CI)** | **positive predictive value:**  **% (95% CI)** | **negative predictive value:**  **% (95% CI)** |
| --- | --- | --- | --- | --- | --- |
|  | **AHI≥5 (73% prevalence)** | | | | |
| TASC≥2 | 72 (58−83) | 74 (52−90) | 0.41 (0.21−0.61) | 87 (74−95) | 52 (34−69) |
| TASC≥3 | 46 (33−60) | 91 (72−99) | 0.28 (0.13−0.44) | 93 (76−99) | 42 (28−57) |
| TASC≥4 | 21 (11−35) | 96 (78−100) | 0.11 (0.01−0.22) | 92 (62−100) | 35 (23−48) |
|  | **AHI≥15 (37% prevalence)** | | | | |
| TASC≥2 | 93 (77−99) | 61 (46−74) | 0.48 (0.31−0.64) | 57 (42−72) | 94 (80−99) |
| TASC≥3 | 74 (54−89) | 86 (73−94) | 0.60 (0.41−0.79) | 74 (54−89) | 86 (73−94) |
| TASC≥4 | 36 (18−57) | 94 (83−99) | 0.34 (0.13−0.56) | 75 (43−95) | 75 (62−85) |
|  | **AH ≥30 (15% prevalence)** | | | | |
| TASC≥2 | 92 (62−100) | 47 (35−60) | 0.18 (0.05−0.30) | 23 (12−38) | 97 (84−100) |
| TASC≥3 | 92 (62−100) | 75 (63−85) | 0.44 (0.24−0.64) | 41 (22−61) | 98 (89−100) |
| TASC≥4 | 58 (28−85) | 92 (82−97) | 0.50 (0.24−0.77) | 58 (28−85) | 92 (82−97) |

OSA: Obstructive Sleep Apnoea. AHI: Apnoea-Hypopnoea Index. AASM: American Academy of Sleep Medicine.

TASC (Trøndelag Apnoea Score): a seven-item OSA proxy with one potential point for each item:

loud and embarrassing snoring (according to others) “mostly/at least three times a week”; breathing pauses during the night (according to others) “mostly/at least three times a week”; restricted daytime activities (spare time, school or job) “mostly/at least three times a week”; hypertension; BMI≥30; age≥50 years; male gender.
